# ASSESSING CHANGE IN SOCIAL CONNECTION: DESIGN CONSIDERATIONS FOR CLINICAL TRIALS WITH OLDER ADULTS

**DOI:** 10.1093/geroni/igae098.1878

**Published:** 2024-12-31

**Authors:** Kimberly Van Orden

**Affiliations:** University of Rochester Medical Center, Rochester, New York, United States

## Abstract

Social disconnection is associated with suicide ideation, attempts, and deaths in later life and is present in 70-90% of suicide deaths in older adults. However, it is not known if promoting social connection is an effective strategy to prevent suicide in later life because studies have not directly tested this mechanism. This presentation will describe the rationale and design of an ongoing clinical trial to test a brief behavioral intervention to increase social connection and thereby reduce suicide risk in older adults in senior living communities who endorse significant loneliness and recent suicide ideation. All study outcomes are measured via smartphone: two subjective indices of social connection are measured via EMA self-report (loneliness and satisfaction with social activities) as well as two objective indices of social connection (time spent outside the home [self-report via EMA, supplemented with GPS sensing] and quantity of conversations [ambient audio sensing]. Suicide ideation is assessed daily via EMA. Findings from baseline smartphone assessments will be presented, including compliance to the protocol. Currently, 5 subjects have completed the 10-day smartphone assessment protocol (2 males, 3 females), with an average age of 88 (range 77-96 years). Few studies with older adults use this methodology for outcomes assessment and no studies have used smartphone assessments to collect outcomes data for behavioral interventions for social connection, despite potential benefits of assessing social connection within real-world social contexts. Opportunities (maximizing sensitivity) and challenges (technology support) to this approach will be discussed.

